# The Relationships between Maternal Feeding Practices and Food Neophobia and Picky Eating

**DOI:** 10.3390/ijerph17113894

**Published:** 2020-05-31

**Authors:** Hebah Alawi Kutbi

**Affiliations:** Clinical Nutrition Department, Faculty of Applied Medical Sciences, King Abdulaziz University, P.O. Box 80215, Jeddah 21589, Saudi Arabia; hkutbi@kau.edu.sa

**Keywords:** food neophobia, picky eating, children, feeding practices, eating behaviors, Saudi Arabia

## Abstract

Food neophobia and picky eating (FNPE) are dietary behaviors that have been frequently reported to coexist in children. Parental concerns about these dietary behaviors may influence the feeding practices employed. In this cross-sectional study, we investigated the bidirectional associations of maternal feeding practices with children’s FNPE behaviors. Using a convenience sampling technique, mothers of 195 healthy children aged 1–7 years were invited to complete a sociodemographic questionnaire, rate their child’s FNPE, and rate the extent to which each feeding practice was employed with the child. Maternal reports indicated that 37.4% (*n =* 73) of the children exhibited severe FNPE. Multiple linear regression analyses showed positive two-way associations between the “pressure to eat” feeding strategy and FNPE, and negative two-way associations between a healthy home food environment and FNPE. However, maternal practices of teaching and monitoring were not found to be associated with FNPE. Given the bidirectional relationships observed between FNPE and maternal feeding practices, primary health care providers should address the feeding practices used with a child and indicate that coercive feeding practices are counterproductive. Intervention studies targeting mothers of children with FNPE are needed to investigate whether specific maternal practices are more effective than others.

## 1. Introduction

Food preferences are typically established during early childhood [1] and may persist into later life [2]. These preferences are important determinants of food choices and dietary quality [1]. Parental feeding practices are known to play a vital role in shaping children’s dietary preferences, behaviors, and attitude toward foods [3,4]. Parents who are anxious about their child not eating enough or perceive the child as too thin may try to employ different strategies to increase the child’s food intake, although these strategies do not always work as intended [5]. Children may resist eating more food or adopt a behavior of excessive food intake that may continue into adulthood, exposing them to the risk of becoming overweight or developing obesity [6]. In fact, several studies in the past suggest that the parental feeding style affects children’s dietary behaviors and weight status [7,8].

Food neophobia and picky eating (FNPE), described as the reluctance of eating novel foods [9] and the rejection of substantial amounts of familiar and unfamiliar foods [10], respectively, are eating behaviors that have been frequently reported to co-exist in children [11,12]. In fact, FNPE might share a common etiological pathway, indicated by the influence of intrinsic and extrinsic factors such as the home environment [11]. However, the associations they may have with sociodemographic factors and maternal feeding behaviors are not necessarily to be the same [12].

Excessive FNPE can be problematic, as a limited variety of food options may affect children’s nutritional status and wellbeing and expose them to a high risk of nutrition-related problems. For example, to meet the nutrient requirements for growth and development, children should consume sufficient quantity of calories and nutrients. Insufficient caloric intake may affect their rate of growth [13]; on the other hand, children who try to avoid healthy foods, such as fruits or vegetables, may consume foods they find more acceptable, such as energy-dense foods, which may increase their risk of obesity and associated comorbidities [14]. Furthermore, FNPE may possibly cause distortions of nutrient intake, such as iron, zinc, and dietary fiber (associated with a low intake of meat, fruits, and vegetables). Without early appropriate interventions, these eating behaviors may persist from childhood to adulthood [15].

Parental feeding practices and children’s eating behaviors have been proposed to have reciprocal effects [8,16]. Parents are often concerned about their child being picky or food neophobic and may use different strategies to compensate for these specific behaviors [17]. On the other hand, the feeding practices employed by parents may inadvertently increase the levels of their child’s FNPE. For example, parents may adopt the “pressure to eat” parenting style to increase the willingness of their child to eat foods that they often reject [8]. Studies have also shown that mothers of children with high levels of food neophobia (FN) tend to use more food restrictions to encourage a healthy lifestyle and less monitoring of food intake and food choices [18]. However, the reverse pathways were found to be inconsistent. Maternal practice of pressuring to eat predicted higher scores of FNPE in several studies [8,12]; another study found no association between maternal pressure and picky eating (PE), but a monitoring practice was found to be inversely associated with PE [19].

In Saudi Arabia as well as in many Arab countries, mothers are often responsible for feeding their children. Therefore, the majority of the studies conducted in these countries have mainly focused on maternal feeding practices rather than on paternal roles [20,21]. However, studies that have investigated the associations between maternal feeding practices and FNPE in Saudi Arabia are particularly lacking. To date, there has been one study that examined the associations between selected maternal feeding practices and FNPE. The study suggested positive associations between the “pressure to eat” feeding strategy and parental modeling and PE, whereas a negative association with repeated exposure was observed [12]. No studies have yet examined the bidirectional relationships between maternal feeding practices and FNPE.

Given that childhood eating behaviors predict eating behaviors in adulthood [15], mothers should be advised on how to appropriately feed their child in a healthy manner that might aid in overcoming or ameliorating negative eating behaviors. Understanding the association of the employed feeding practices with FNPE must be established to develop evidence-based recommendations and design effective interventions. With the primary aim of filling the gap in the literature, the present study aimed to examine the bidirectional associations between FNPE and maternal feeding practices, including pressure feeding, teaching, monitoring, and healthy home food environment, in a sample of children and their mothers in Saudi Arabia.

## 2. Materials and Methods

### 2.1. Sample and Procedures

This was a cross-sectional study of healthy children residing in the Kingdom of Saudi Arabia. Mothers of children aged between one to seven years with no food allergy were recruited conveniently through the social media platforms Twitter and WhatsApp, informed about the objectives of the study, and invited to complete an online questionnaire. The participants were also informed that the information provided would remain confidential and anonymous. The sample included data of 234 participants collected between September and October 2019. However, we excluded data of participants reporting residency in a country other than Saudi Arabia and data of children outside the targeted age range (*n =* 29). Additionally, each of 10 mothers reported data of 2 children; the data of the older sibling were excluded from the analyses. The final analyses included data of 195 participants. The required sample size was estimated based on a correlation coefficient of 0.25, the average correlation between FNPE and maternal feeding strategies of a previously reported study [12], with a 2-sided test, alpha of 0.05, and power of 95% [22]. The Ethics and Research Committee, Faculty of Applied Medical Sciences, King Abdulaziz University, approved the study.

### 2.2. Measures

#### 2.2.1. Demographic Variables

The questionnaire included questions about the city of residence (later grouped into Western, Central, and other regions), nationality (Saudi vs. not Saudi), maternal age (later grouped into <30, 30–39, and ≥40 years old), maternal education (less than college degree vs. college degree or higher), maternal working status (not working vs. working), and child’s sex. The child’s age was calculated based on the reported date of birth and grouped into less than 2, 2–4, and 5–7 years.

#### 2.2.2. FNPE Scales

The degree of children’s FN was assessed using the Food Neophobia Scale (FNS), a 10-item tool [9]. The mean score of the 10 items was calculated, wherein the items were to be rated on a scale of 1 (strongly disagree) to 7 (strongly agree), and a rating of 7 indicated the highest level of FN [9]. The FNS in the present study demonstrated high internal consistency, Cronbach’s alpha = 0.86.

Levels of PE among children in our sample were measured using the PE subscale (3 items) of the Child Eating Behavior Questionnaire (CEBQ). The mean score of the three items was calculated and used to indicate the extent to which PE was exhibited by a child, wherein the items were to be rated on a scale of 1 (disagree) to 5 (agree), and a rating of 5 indicated the highest level of PE [10]. The PE scale in our sample showed good internal consistency, Cronbach’s α = 0.76.

#### 2.2.3. Maternal Feeding Practices Scales: Pressure to Eat, Teaching, Monitoring, and Providing a Healthy Home Food Environment

The feeding practice scales of pressure to eat, teaching, monitoring, and healthy home food environment were adapted from the Comprehensive Feeding Practices Questionnaire (CFPQ). Each subscale was rated by the mothers on a 5-point Likert scale [23]. The CFPQ has been previously validated in the Middle East [20].

Maternal use of the pressure feeding strategy was assessed using the statements: “My child should always eat all of the food on his/her plate”; “If my child says, ‘I’m not hungry,’ I try to get him/her to eat anyway”; “If my child eats only a small amount of food, I try to get him/her to eat more”; and “When he/she says he/she is finished eating, I try to get my child to eat one more (two more, etc.) bite of food.” The mean score of the items was calculated, wherein the items were to be rated on a scale of 1 (disagree) to 5 (agree), and a rating of 5 indicated a greater use of pressure feeding (Cronbach’s α = 0.67). The item “My child should always eat all of the food on his/her plate” was later deleted to improve the internal consistency of the scale (Cronbach’s α = 0.78).

Maternal practice of teaching was assessed using the statements: “I discuss with my child why it’s important to eat healthy foods”; “I discuss with my child the nutritional value of foods”; and “I tell my child what to eat and what not to eat without explanation” (reverse-coded). The response options ranged from 1 (disagree) to 5 (agree) (Cronbach’s α = 0.72). The abovementioned third item was later deleted to enhance the internal consistency of the scale (Cronbach’s α = 0.89). The mean score was calculated to indicate the extent to which mothers were using the teaching technique when feeding their child, wherein 5 indicated a greater use of the teaching technique.

Monitoring the feeding practice was evaluated using the questions: “How much do you keep track of sweets (candies, ice cream, cake, pies, pastries) that your child eats?”; “How much do you keep track of snacks (potato chips, Doritos, cheese puffs) that your child eats?”; “How much do you keep track of high-fat foods that your child eats?”; and “How much do you keep track of sugary drinks (soda/pop) that your child drinks?”. The items were rated on a scale of 1 (never) to 5 (always). The average score indicated the degree of maternal monitoring of their child’s food consumption, wherein 5 indicated the greatest degree of monitoring (Cronbach’s α = 0.86).

The scale assessing provision of a healthy home food environment included the statements: “Most of the food I keep in the house is healthy”; “I keep a lot of snacks (potato chips, Doritos, cheese puffs) in my house” (reverse coded); “A variety of healthy foods are available to my child at each meal served at home”; and “I keep a lot of sweets (candies, ice cream, cake, pies, pastries) in my house” (reverse-coded). Each item was rated on a scale of 1 (disagree) to 5 (agree), and the mean score of the items was calculated to indicate the extent to which mothers were providing a healthy home food environment (Cronbach’s α = 0.71).

### 2.3. Statistical Analysis

Descriptive data were illustrated as frequency, percentages, mean, and standard deviation (SD). FN, but not PE, was found to be normally distributed. Therefore, we examined the associations between the sociodemographic variables and FN using Student’s t-test and one-way ANOVA, while the associations with PE were examined using Mann–Whitney and Kruskal–Wallis tests. The associations of the maternal feeding practices, including the pressure to eat, providing a healthy home food environment, teaching, and monitoring, with the sociodemographic variables were also investigated using Mann–Whitney and Kruskal–Wallis tests. To explore the prevalence of FNPE, we categorized the two variables according to the mean scores into 3 groups [24,25]: slight, moderate, and severe. The intercorrelation between the continuous variables was examined using Spearman’s rank correlation coefficients. For all the analyses, *p* < 0.05 was used to determine statistical significance. No multicollinearity issue was detected; therefore, we used linear regression models and 95% confidence intervals [95% CIs] to investigate the associations between FNPE and each maternal feeding strategy. Multiple linear regression models were adjusted for child’s and maternal age (categorical variables). Statistical analyses were conducted using Statistical Package for Social Sciences (SPSS) version 24.0 (Armonk, NY, USA).

## 3. Results

Sample characteristics are described in Table 1. Children’s age ranged between 1.22 and 6.99 years, with a mean age of 4.14 ± 1.60 years. Maternal age ranged between 21 and 49 years, with a mean age of 33 ± 4.63 years. The data indicated that 10.3% of the children (*n =* 20) exhibited slight FN, whereas 52.3% (*n =* 102) and 37.4% (*n =* 73) exhibited moderate and severe FN, respectively. Additionally, 19.5% of the children (*n =* 38) exhibited slight PE, whereas 43.1% (*n =* 84) and 37.4% (*n =* 73) exhibited moderated and severe PE, respectively. Univariate analyses were performed to investigate the associations between the sociodemographic variables and FN and PE. The mean scores of FN and PE were statistically significantly different across the maternal age groups, wherein the mean scores of FN in children of mothers aged less than 30, 30–39, and ≥40 years old were 4.55 ± 1.38, 3.99 ± 1.34, and 4.62 ± 1.27, respectively (*p* = 0.018), and the mean scores of PE were 3.30 ± 1.11, 2.86 ± 1.14, and 3.32 ± 1.26, respectively (*p* = 0.035).

The associations between maternal feeding practices and the sociodemographic characteristics were also investigated. The mean scores of the teaching strategy were found to be significantly different across child’s age groups. The mean scores of the teaching scale for children’s aged <2, 2–5, and ≥5 years were 3.03 ± 1.23, 3.66 ± 1.31, and 4.17 ± 1.07, respectively (*p* = 0.001).

The intercorrelation between FNPE and maternal feeding strategies are presented in Table 2. FN and PE were found to be strongly correlated (r_s_ = 0.760, *p* < 0.001). Positive correlations were observed between the pressure feeding strategy and both FN (r_s_ = 0.200, *p* = 0.005) and PE (r_s_ = 0.185, *p* = 0.010), whereas a negative correlation was observed between a healthy home food environment and PE (r_s_ = −0.195, *p* = 0.006). Furthermore, teaching, monitoring, and healthy home food environment practices were found to be positively correlated (*p* < 0.001), but none was correlated with the “pressure to eat” strategy (*p* > 0.050).

The associations between maternal feeding practices and FNPE are presented in Table 3. Multiple linear regression analyses controlled for maternal and child’s ages showed positive two-way associations between the “pressure to eat” strategy and FNPE. Maternal practice of pressure feeding predicted higher levels of FN (B = 0.21 [95% CI: 0.05 to 0.37]) and PE (B = 0.17 [95% CI: 0.03 to 0.31]). The reverse associations were also observed, wherein higher scores of FN and PE predicted a greater practice of pressure feeding (B = 0.16 [95% CI: 0.04 to 0.28] and B = 0.18 [95 CI: 0.03 to 0.32], respectively).

On the other hand, providing a healthy home food environment showed a negative two-way association with FNPE. The maternal practice of providing a healthy home food environment predicted lower levels of FN (B= −0.23 [95% CI: −0.45 to −0.02]) and PE (B= −0.28 [95% CI= −0.46 to −0.09]). Likewise, FN and PE predicted a less frequent practice of providing a healthy food environment (B = −0.10 [95% CI: −0.19 to −0.01] and B = −0.16 [95% CI: −0.27 to −0.05], respectively). However, no association between maternal practice of teaching or monitoring with FN or PE was observed.

## 4. Discussion

Maternal concerns about children’s dietary behaviors and nutritional status may influence the kind of feeding practices employed [8,26]. This study investigated the associations of maternal feeding practices, including pressure in feeding, teaching, monitoring, and healthy home food environment, with children’s FNPE behaviors. Our results suggested bidirectional relationships between the “pressure to eat” strategy and a healthy home food environment and FNPE. Positive two-way associations between maternal practice of pressure feeding and FNPE were observed. Additionally, the higher tendency to provide a healthy home food environment was associated with lower scores on FNPE, whereas FNPE in children predicted a lower tendency of the mothers to provide a healthy home food environment.

Our findings are aligned with that of Jansen et al., wherein the association of FNPE with pressure feeding practice is bidirectional [8]. Mothers are possibly concerned about their children being picky or unwilling to try new foods, eliciting the use of pressure feeding to enforce healthier eating habits and practices [27]. A twin study conducted to investigate differences in maternal feeding practices used with their children with differing levels of PE found that mothers practiced greater pressure on the child perceived as being pickier than on the co-twin [28]. However, the current evidence suggests that coercive feeding is counterproductive. A study conducted in Saudi Arabia indicated that the pressure feeding practice is associated with greater severity of FNPE [12]. Another study has also reported that using pressure in feeding at the age of 4 predicted higher levels of PE in children at 6 years of age [8].

Previous work has recommended alternating the pressure feeding strategy with other feeding practices to improve a child’s acceptance of foods [8]. In fact, existing evidence suggests that parental modelling and food exposure may improve healthy food preferences [29]. However, feeding practices that could enhance children’s acceptance of foods among preschoolers in Saudi Arabia are still unidentified. In the present study, the use of teaching, monitoring, and healthy home food environment practices were found to be intercorrelated, indicating that mothers are making an effort to improve their children’s liking of foods using different feeding strategies. However, it is important to identify which of these strategies is possibly effective. Our findings indicated that providing a healthy home food environment is negatively associated with FNPE in children, while no such association was observed with the maternal use of teaching or monitoring practices. These results are aligned with previously reported findings. For instance, Shim et al. [30] investigated the associations of parental feeding practices with fruit and vegetable intake in preschoolers; they found a significantly positive relationship between a healthy home food environment and the consumption of vegetables. This finding indicates that picky eaters tend to improve their acceptance of food if they are exposed to a healthy home food environment. On the other hand, a quasi-experimental study examined whether school-based nutrition education improves taste acceptance and healthy eating of elementary school children. The authors observed a significant improvement in knowledge; however, the effect on children’s acceptance of novel foods was marginal, possibly due to the short duration of the experiment (6 months) [31]. Another intervention study of children aged between 4 and 8 years investigated the associations of teaching and monitoring practices with PE, and no association was observed [32]. Even though we were unable to detect any association between teaching or monitoring practices and FNPE behaviors, we hypothesize that monitoring and teaching strategies may enhance children’s knowledge of healthy food options and may possibly improve their acceptance of foods in later childhood. However, future prospective studies are necessary to examine the effects of these practices in later childhood.

Our data also showed that FNPE in children predict a lower tendency of mothers to provide a healthy food environment. Maternal concern about their child not eating sufficient amounts of food or having limited food preferences may cause them to increase accessibility to foods their children like, whether healthy or unhealthy, to compensate for the limited food intake. However, these actions may encourage the children to adopt unhealthy dietary habits that may last to adulthood [15]; this may also increase the risk of obesity and associated comorbidities, if children mainly rely on calorie-dense foods or have a limited intake of fruits and vegetables [14].

Previous studies investigated the association between maternal age and FNPE. A study reported a negative association between maternal age and PE [33]; another study did not observe an association with FN [34]. Our data rather suggest that maternal age and FNPE might present an inverted U-shaped curvilinear association. The mean scores of FN were statistically significantly higher for children of mothers aged <30 years (4.55 ± 1.38) and mothers aged ≥40 years (4.62 ± 1.27) than for children of mothers aged 30–39 years (4.62 ± 1.27). Similarly, the mean scores of PE were significantly higher for children of mothers aged <30 years (3.30 ± 1.11) and mothers aged ≥40 years (3.32 ± 1.26) than for children of mothers aged 30–39 years (2.86 ± 1.14).

The present study has several strengths. In Saudi Arabia, we were able to identify only one study that explored the association of FNPE with environmental and cognitive factors in children in Jeddah city. Therefore, our analysis responds to the need for evidence-based literature looking at the associations of specific maternal feeding practices with FNPE to establish effective interventions. The adequate sample size and power allowed us to detect significant associations. However, our study is limited by the nature of its design as a cross-sectional study; we were unable to infer any cause-and-effect relationship. Additionally, the sample of this study was conveniently recruited and only included mothers with internet access and who use the social media. Further, the data were based on self/mother-reported measures; therefore, social desirability and recall bias are possible. Mothers perceiving their child as too thin may be overestimating the child’s PE. To overcome this limitation, children could be observed during mealtimes to better estimate their levels of FNPE behaviors.

## 5. Conclusions

Our findings indicate bidirectional relationships between FNPE in children and maternal feeding practices of pressure and of providing a healthy home food environment. Primary health care providers should address the feeding practices used with a child and indicate that the coercive feeding practice might be ineffective in promoting children’s consumption of healthy foods [7]. Our results may be used to establish intervention studies seeking to improve children’s acceptance of foods and to investigate whether specific maternal practices are effective or more useful than others. Further, longitudinal studies on the effect of teaching and monitoring on children’s acceptance of food in the long term may possibly reveal a beneficial effect in later childhood.

## Figures and Tables

**Table 1:** Characteristics of the sample.

Variable		*n =* 195
Region, *n* (%)	Western region	155 (79.5)
	Central region	22 (11.3)
	Other regions	18 (9.20)
Child’s sex, *n* (%)	Male	101 (51.8)
	Female	94 (48.2)
Child’s age in years, *n* (%)	Less than 2	18 (9.20)
	2–4	108 (55.4)
	5–7	69 (35.4)
Household income in Saudi Arabian Riyals, *n* (%)	Less than 5000	18 (9.20)
	5000–<10,000	46 (23.6)
	10,000–<15,000	51 (26.2)
	15,000–20,000	34 (17.4)
	Over 20,000	46 (23.6)
Saudi nationality, *n* (%)	Yes	176 (90.3)
	No	19 (9.70)
Maternal age in years, *n* (%)	Less than 30	45 (23.1)
	30–39	131 (67.2)
	≥40	19 (9.70)
Maternal education, *n* (%)	Less than college degree	34 (17.4)
	College degree or higher	161 (82.6)
Maternal working status, *n* (%)	Not working	75 (38.5)
	Working	120 (61.5)
Food neophobia, mean (SD) *		4.18 (1.37)
Picky eating, mean (SD) ^†^		3.01 (1.16)
Pressure, mean (SD) ^†^		2.76 (1.20)
Healthy home food environment, mean (SD) ^†^		3.52 (0.88)
Teaching, mean (SD) ^†^		3.78 (1.26)
Monitoring, mean (SD) ^†^		4.08 (0.92)

* 7-point scale. ^†^ 5-point scale.

**Table 2:** Intercorrelations among measures of food neophobia, picky eating, and maternal feeding practices using Spearman correlation matrix (*n =* 195).

Variable		Food Neophobia	Picky Eating	Pressure to Eat	Healthy Home Food Environment	Teaching	Monitoring
Food neophobia	r	--	0.760 ^†^	0.200 ^†^	−0.129	−0.016	−0.082
	*p*-value		<0.001	0.005	0.071	0.830	0.252
Picky eating	r	0.760 ^†^	--	0.185 ^†^	−0.195 ^†^	−0.019	−0.049
	*p*-value	<0.001		0.010	0.006	0.790	0.495
Pressure to eat	r	0.200 ^†^	0.185 ^†^	--	−0.138	0.051	−0.131
	*p*-value	0.005	0.010		0.055	0.478	0.068
Healthy home food environment	r	−0.129	−0.195 ^†^	−0.138	--	0.294 ^†^	0.500 ^†^
	*p*-value	0.071	0.006	0.055		<0.001	<0.001
Teaching	r	−0.016	−0.019	0.051	0.294 ^†^	--	0.385 ^†^
	*p*-value	0.830	0.790	0.478	<0.001		< 0.001
Monitoring	r	−0.082	−0.049	−0.131	0.500 ^†^	0.385 ^†^	--
	*p*-value	0.252	0.495	0.068	<0.001	< 0.001	
Child’s age	r	0.148 *	0.071	0.145 *	−0.009	0.279 ^†^	−0.024
	*p*-value	0.039	0.324	0.043	0.899	< 0.001	0.739
Maternal age	r	−0.054	−0.077	−0.006	0.081	0.183 ^†^	0.047
	*p*-value	0.451	0.284	0.938	0.258	0.010	0.514

* Significant at the 0.05 level (2-tailed). ^†^ Significant at the 0.01 level (2-tailed).

**Table 3:** Relationships of food neophobia and picky eating with maternal feeding practices.

Predictor/Outcome Variable	B Estimate	95% CI *	*p*-Value	B Estimate	95% CI	*p*-Value
	Unadjusted Model			Adjusted Model ^§^		
Pressure to eat/FN ^†^	0.22	0.06, 0.38	0.007	0.21	0.05, 0.37	0.011
Teaching/FN	−0.05	−0.20, 0.11	0.535	−0.09	−0.25, 0.07	0.256
Monitoring/FN	−0.17	−0.38, 0.04	0.106	−0.16	−0.37, 0.05	0.127
Healthy home food environment/FN	−0.23	−0.45, −0.02	0.037	−0.23	−0.45, −0.02	0.035
Pressure to eat/PE ^‡^	0.17	0.04, 0.31	0.013	0.17	0.03, 0.31	0.016
Teaching/PE	−0.04	−0.17, 0.10	0.602	−0.05	−0.19, 0.09	0.464
Monitoring/PE	−0.05	−0.22, 0.13	0.622	−0.04	−0.22, 0.14	0.673
Healthy home food environment/PE	−0.28	−0.46, −0.09	0.003	−0.28	−0.46, −0.09	0.003
FN/Pressure to eat	0.17	0.05, 0.29	0.007	0.16	0.04, 0.28	0.011
FN/Teaching	−0.04	−0.17, 0.09	0.535	−0.07	−0.20, 0.05	0.256
FN/Monitoring	−0.08	−0.17, 0.02	0.106	−0.08	−0.17, 0.02	0.127
FN/Healthy home food environment	−0.10	−0.19, −0.01	0.037	−0.10	−0.19, −0.01	0.035
PE/Pressure to eat	0.18	0.04, 0.33	0.013	0.18	0.03, 0.32	0.016
PE/Teaching	−0.04	−0.20, 0.11	0.602	−0.06	−0.20, 0.09	0.464
PE/Monitoring	−0.03	−0.14, 0.09	0.622	−0.02	−0.14, 0.09	0.673
PE/Healthy home food environment	−0.16	−0.26, −0.05	0.003	−0.16	−0.27, −0.05	0.003

Abbreviations: * CI, confidence interval ^†^ FN, food neophobia; ^‡^ PE, picky eating. ^§^ Adjusted for maternal and child’s ages.
